# Identification and Management of Intraoperative Pneumothorax During Laparoscopic Surgery: A Rare Complication

**DOI:** 10.7759/cureus.106113

**Published:** 2026-03-30

**Authors:** Feras M Al-Majmoai

**Affiliations:** 1 Anesthesiology and Critical Care, Rashid Hospital, Mohammed Bin Rashid University (MBRU), Dubai, ARE

**Keywords:** a rare complication of laparoscopic surgery, identification of intraoperative pneumothorax, management of intraoperative pneumothorax, pneumothorax as a complication of laparoscopic surgery, pneumothorax during laparoscopic surgery

## Abstract

Pneumothorax is a rare but potentially life-threatening intraoperative complication during general anesthesia, with early recognition often challenging due to nonspecific hemodynamic and respiratory changes that may mimic other anesthetic or surgical issues. The risk is increased in laparoscopic procedures due to pneumoperitoneum and possible diaphragmatic or pleural injury, as well as in the presence of preexisting pulmonary disease, elevated airway pressures, central venous catheterization, and airway manipulation during intubation. Although it is an uncommon complication in laparoscopic surgeries, delayed diagnosis can lead to hypoxia, hemodynamic instability, and fatal outcomes. We report a case of a 34-year-old female patient (ASA II) with superior mesenteric artery syndrome and a history of gastrojejunostomy who underwent emergency duodenojejunostomy with reversal of the gastrojejunostomy under general anesthesia. The patient was intubated successfully and ventilated using pressure-controlled ventilation; however, intraoperatively, airway pressures rose to 29 cmH₂O, and capnography showed a delayed upstroke, indicating possible airflow obstruction. She developed bilateral subcutaneous emphysema over the neck, oxygen desaturation to 85%, tachycardia (120 bpm), hypertension (mean arterial pressure 119 mmHg), and elevated end-tidal CO₂ (>55 mmHg). The surgery was immediately halted, endotracheal tube placement was confirmed, and manual ventilation with 100% oxygen improved oxygen saturation to above 93%. Reduced air entry over the right lung field raised suspicion of right-sided pneumothorax, which was confirmed via intraoperative chest radiography. An emergency intercostal chest tube was inserted, leading to rapid improvement in ventilation and oxygenation. The procedure was subsequently aborted, and the patient was transferred to the intensive care unit for monitoring. She recovered without complications, was discharged in stable condition, and remained asymptomatic on follow-up without recurrence. This case highlights the critical importance of vigilance, early recognition of warning signs, and prompt intervention to prevent severe complications.

## Introduction

Pneumothorax is a rare, potentially lethal intraoperative complication [1,2]. Early recognition during general anesthesia is challenging, as hemodynamic variations are usually nonspecific. Risk factors include the presence of emphysematous bullae or blebs, surgical manipulation of areas close to the parietal pleura, central venous line placement, and pneumoperitoneum [1-4]. Its incidence during laparoscopic surgery is 0.4% [5]. Rarely, it is associated with airway manipulation, particularly when other risk factors are present. Recognition is difficult intraoperatively and may only be identified postoperatively [1-4]. This case highlights the management of a pneumothorax that was discovered during laparoscopic surgery.

## Case presentation

**Figure 1:**
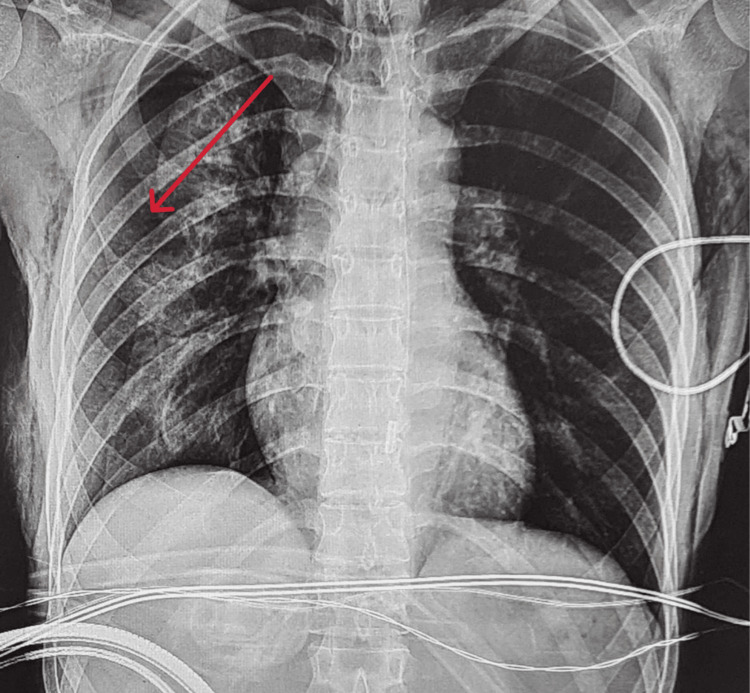
Intraoperative X-ray (AP view in the supine position) confirming the presence of right-sided pneumothorax

A 34-year-old woman with superior mesenteric artery syndrome and prior gastrojejunostomy presented with abdominal pain and persistent vomiting. She was scheduled for emergency duodenojejunostomy with reversal of the gastrojejunostomy under general anesthesia (ASA-2) [6]. Anesthesia was induced with intravenous propofol, ketamine, fentanyl, and rocuronium and maintained with desflurane and dexmedetomidine infusion. The patient was successfully intubated, and endotracheal tube placement was confirmed by auscultation. She was ventilated using pressure-controlled ventilation. Intraoperatively, airway pressure gradually increased to over 29 cmH₂O, and the capnogram showed a delayed upstroke, suggesting airway obstruction. Subcutaneous emphysema was palpated bilaterally over the neck. Oxygen saturation (SpO_2_) decreased to 85%, accompanied by tachycardia (120 beats per minute), hypertension (mean arterial pressure 119 mmHg), and elevated end-tidal carbon dioxide > 55 mmHg. The anesthesiologist immediately suspended the procedure, confirmed the patency and placement of the endotracheal tube, and manually ventilated with 100% oxygen, restoring SpO_2_ to above 93%. Auscultation revealed decreased air entry over the right lung, raising suspicion of right- sided pneumothorax, which was confirmed by an immediate intraoperative chest radiography (Figure 1). An emergency intercostal chest tube was inserted, resulting in rapid improvement in ventilation and oxygenation. The surgery was subsequently aborted, and the patient was transferred to the intensive care unit for close monitoring. Her postoperative course was stable and uneventful, and she was later discharged in good condition, remaining asymptomatic at follow-up without pneumothorax. Laboratory and intraoperative monitoring parameters are summarized in Table 1.

**Table 1:** Laboratory and intraoperative monitoring parameters SpO₂: Peripheral capillary oxygen saturation; MAP: Mean arterial pressure

Parameter	Patient Value	Reference Range
SpO₂ (%)	85	95–100
End-tidal carbon dioxide (mmHg)	> 55	35–45
Heart rate (beats per minute)	120	60–100
MAP (mmHg)	119	70–100
Airway pressure (cmH₂O)	> 29	10–20

## Discussion

Pneumothorax during general anesthesia is a rare but potentially life-threatening complication, particularly in the setting of positive pressure ventilation, where even minimal pleural disruption may rapidly progress to tension physiology [1-4]. Although uncommon, it carries significant morbidity due to the risk of acute hypoxemia, hemodynamic instability, and cardiovascular collapse. Closed claims analyses have shown that pneumothorax accounts for a small proportion of perioperative respiratory events but is associated with poor outcomes, including death or severe neurological injury when diagnosis is delayed [1].

The pathophysiology is multifactorial. Positive pressure ventilation increases alveolar distending pressure, predisposing to alveolar rupture. In laparoscopic surgery, pneumoperitoneum from CO₂ insufflation elevates intra-abdominal pressure and may result in diaphragmatic defects or pleuroperitoneal communication, allowing gas to enter the pleural space [2,4,7]. This may lead to capnothorax, which can present with rapid hemodynamic compromise due to increased intrathoracic pressure and reduced venous return, particularly when gas inflow is rapid or a large pleural defect is present [2].

Risk factors include preexisting pulmonary abnormalities such as emphysematous bullae, which may remain undetected on routine chest radiography [1,4]. Mechanical ventilation is a key contributor, with tidal volume, inspiratory flow, and airway pressures influencing the risk of barotrauma. While lung-protective ventilation strategies are commonly used, their role in preventing pneumothorax remains uncertain [1]. The impact of positive end-expiratory pressure is also complex and may reflect underlying lung pathology rather than an independent risk factor [1]. Additional perioperative contributors include airway manipulation during intubation and central venous catheterization, particularly via the subclavian route, which has been associated with a higher incidence of mechanical complications including pneumothorax [3].

Early diagnosis is challenging due to nonspecific clinical features. Sudden increases in airway pressures, reduced lung compliance, hypoxemia, hypercapnia, and hemodynamic instability may mimic bronchospasm, endobronchial intubation, or equipment malfunction [1,4]. In this case, worsening oxygenation with positive pressure ventilation and transient improvement upon disconnection suggested tension physiology. The presence of subcutaneous emphysema and unilateral reduction in breath sounds further supported the diagnosis. However, differentiation from conditions such as bronchospasm or air-trapping remains difficult, as these may produce similar findings and can themselves lead to barotrauma [1].

Management depends on severity. Immediate measures include administration of 100% oxygen, reduction of airway pressures, and prompt communication with the surgical team [1,4]. In laparoscopic procedures, desufflation of pneumoperitoneum is essential. In suspected tension pneumothorax, emergent decompression followed by intercostal chest tube insertion is the standard of care [4]. In contrast, selected cases of capnothorax without significant instability may be managed conservatively due to rapid CO₂ absorption [2]. In the present case, prompt recognition and chest tube insertion resulted in rapid clinical improvement.

This case highlights the importance of vigilance and structured crisis management during anesthesia. Preoperative evaluation, especially with chest CT scan and spirometry, is very important. Early recognition using a structured crisis algorithm, effective team communication, and prompt imaging allowed rapid intervention. An intercostal tube was inserted to stabilize the patient, after which the laparoscopic surgery was safely aborted. Timely management is critical regardless of the underlying cause of pneumothorax.

## Conclusions

Pneumothorax is a rare but potentially fatal complication. Early recognition and management can prevent life-threatening consequences. Utilization of intraoperative monitoring and access to diagnostic tools are essential to facilitate early diagnosis and effective intervention.
